# Case-Control Cohort Study of Patients' Perceptions of Disability in Mastocytosis

**DOI:** 10.1371/journal.pone.0002266

**Published:** 2008-05-28

**Authors:** Olivier Hermine, Olivier Lortholary, Phillip S. Leventhal, Adeline Catteau, Frédérique Soppelsa, Cedric Baude, Annick Cohen-Akenine, Fabienne Palmérini, Katia Hanssens, Ying Yang, Hagay Sobol, Sylvie Fraytag, David Ghez, Felipe Suarez, Stéphane Barete, Philippe Casassus, Beatrice Sans, Michel Arock, Jean Pierre Kinet, Patrice Dubreuil, Alain Moussy

**Affiliations:** 1 Service de Dermatologie, Centre de référence sur la mastocytose, Hôpital Necker, Paris, France; 2 Service d'Hématologie Adulte, Centre de référence sur la mastocytose, Hôpital Necker, Paris, France; 3 Association Française pour les initiatives et la recherche sur les mastocytes et les mastocytoses (AFIRMM), Paris, France; 4 INSERM UMR599, Centre de Recherche en Cancérologie de Marseille, Laboratoire d'Hématopoïèse Moléculaire et Fonctionnelle, Marseille, France; 5 Service d'anatomopathologie, Centre de référence sur la mastocytose, Hôpital Necker, Paris, France; 6 Département d'Oncologie Génétique, Centre de référence sur la mastocytose, Institut Paoli Calmettes, Marseille, France; 7 Service de Dermatologie, Hôpital Tenon, Université Paris VI, Centre de référence sur la mastocytose, Assistance Publique Hôpitaux de Paris, Paris, France; 8 Service d'hématologie, Hôpital Avicennes, CHU Bobigny, Université Paris XIII, Paris, France; 9 Service de Dermatologie, vénérologie et allergologie, Hôpital Purpan, place du Docteur Baylac, Toulouse, France; 10 Department of infectious and tropical diseases, Centre de référence sur les mastocytoses, Hôpital Necker, Assistance Publique Hôpitaux de Paris, Paris, France; The University of Queensland, Australia

## Abstract

**Background:**

Indolent forms of mastocytosis account for more than 90% of all cases, but the types and type and severity of symptoms and their impact on the quality of life have not been well studied. We therefore performed a case-control cohort study to examine self-reported disability and impact of symptoms on the quality of life in patients with mastocytosis.

**Methodology/Principal Findings:**

In 2004, 363 mastocytosis patients and 90 controls in France were asked to rate to their overall disability (OPA score) and the severity of 38 individual symptoms. The latter was used to calculate a composite score (AFIRMM score). Of the 363 respondents, 262 were part of an ongoing pathophysiological study so that the following data were available: World Health Organization classification, standard measures of physical and psychological disability, existence of the D816V KIT mutation, and serum tryptase level. The mean OPA and AFIRMM scores and the standard measures of disability indicated that most mastocytosis patients suffer from disabilities due to the disease. Surprisingly, the patient's measurable and perceived disabilities did not differ according to disease classification or presence or absence of the D816V KIT mutation or an elevated (≥20 ng/mL) serum tryptase level. Also, 32 of the 38 AFIRMM symptoms were more common in patients than controls, but there were not substantial differences according to disease classification, presence of the D816V mutation, or the serum tryptase level.

**Conclusions:**

On the basis of these results and for the purposes of treatment, we propose that mastocytosis be first classified as aggressive or indolent and that indolent mastocytosis then be categorized according to the severity of patients' perceived symptoms and their impact on the quality of life. In addition, it appears that mastocytosis patients suffer from more symptoms and greater disability than previously thought, that mastocytosis may therefore be under-diagnosed, and that the symptoms of the indolent forms of mastocytosis might be due more to systemic release of mediators than mast cell burden.

## Introduction

Mastocytosis is a disease characterized by the excessive accumulation of mast cells in at least one of several organs, including the skin, bone marrow, lymph nodes, liver, spleen, and gastrointestinal tract [1]–[3]. The true incidence of mastocytosis is unknown, but the available evidence suggests that it is a rare disease, with a prevalence of no more than 0.3 per 10,000, which qualifies it as an orphan disease [4], [5]. In aggressive forms of mastocytosis, the accumulation of mast cells in organs and tissues causes a loss of function and degeneration, which can decrease life expectancy. Aggressive forms of mastocytosis are rare (<10% of all cases) and require specific treatment aimed at reducing mast cell infiltration and activity. Patients with indolent forms of mastocytosis, however, do not have a decreased life expectancy or organ damage, but they can suffer from a very wide variety of signs and symptoms, including pruritus, flushing, syncope, hypotensive shock, dizziness, abdominal pain, nausea, vomiting, diarrhea, fatigue, memory loss, depression, tachycardia, palpitations, breathing difficulties, fractures/osteoporosis, and pain in the muscles, joints, and bones [1]–[3]. These systemic manifestations are believed to be due to the release of mast cell-derived mediators, such as histamine, prostaglandins, heparin, neutral proteases, acid hydrolases, chemokines, and cytokines.

Therefore, mastocytosis is now known to be a multidimensional disease with a wide variety of signs and symptoms. This has complicated its diagnosis, classification, and treatment. In 2001, to help address this problem, the World Health Organization (WHO) developed a consensus classification system for mastocytosis [2]. This system separates mastocytosis into cutaneous mastocytosis (CM) and five main subtypes of systemic mastocytosis (SM), including indolent SM, SM with an associated hematologic clonal, non-mast cell lineage disease, aggressive SM, smoldering SM, and mast cell leukemia. CM is diagnosed by the presence of skin lesions and the absence of definitive systemic involvement by mast cells. In the majority of cases, a diagnosis of SM is established by evidence of mast cell infiltration in the bone marrow or, less frequently, the liver, spleen or gastrointestinal tract. Minor criteria for a diagnosis of SM can include abnormal mast cell morphologies in the bone marrow and other extracutaneous organs, mutation of the tyrosine kinase KIT at codon 816, expression of CD2 and/or CD25 by bone marrow mast cells, and a serum tryptase level >20 ng/mL. CM is most common in children before puberty, most often presents as a rash or urticaria pigmentosa, and often resolves spontaneously. In adults, however, CM frequently progresses to SM. In contrast to CM, SM has a peak onset in adults, tends to be more aggressive, and can involve the skin as well as internal organs and bone marrow. Indolent SM accounts for 90% of the cases of SM and generally appears to have a good prognosis, although a wide variety of mediator-related symptoms are common, and these can be disabling or even life-threatening as in the case of hypotensive shock [3].

In the last 15 years, activating mutations have been found in codon 816 of the tyrosine kinase KIT in children and adults with mastocytosis [6]. In particular, the D816V mutation has been found in most (>80%) of patients with SM and therefore has been thought to promote the development of systemic and persistent disease. Furthermore, a recent study showed that expression of KIT with the D816V mutation causes mastocytosis in transgenic mice [7]. For this reason, inhibitors of the KIT tyrosine kinase are being developed for the treatment of mastocytosis [1].

Since 2001, to help understand the pathophysiology of mastocytosis and improve its treatment, AFIRMM (Association Française pour les Initiatives et la Recherche sur le Mastocyte et les Mastocytoses; http://www.afirmm.com/) has collected data on mastocytosis patients in France. To help physicians in the selection of appropriate treatments for mastocytosis, we performed a case-control study within the AFIRMM network to assess the patients' perception of symptoms and the impact on their quality of life. On the basis of this information, we developed and validated a composite score that measures mastocytosis patients' perception of disability (AFIRMM score). Using this score and an overall rating of self-perceived disability (OPA score), we examined the relationship between disability and the disease classification, the presence of the D816V KIT mutation, and the serum tryptase level. The AFIRMM score also allowed us to identify the symptoms that most contributed to the patients' perception of disability.

## Methods

### The AFIRMM network

The AFIRMM network (http://www.afirmm.com/) was created in France in 1999 to collect data on patients suffering from mastocytosis, inform health care professionals and patients about mast cell disorders, support research to better understand mast cell disorders and thereby develop efficient symptomatic or curative treatments. The AFIRMM network currently includes more than 68 hospitals and clinical centers in France and Switzerland (principal investigators O.H. and O.L., Centre de référence de mastocytose, Hôpital Necker, Paris, France). Between 1999 and 2004, 1297 adult mastocytosis patients were identified in France by AFIRMM.

### Study design and objectives

A case-control study was initiated in September 2004 by AFIRMM to examine patients' disability due to mastocytosis. The objectives of the current cohort study were to (i) evaluate the patients' perception of disability, (ii) establish and validate a composite score for disability, (iii) determine the most important symptoms causing the patients' perception of disability, and (iv) correlate disability with mastocytosis classification and presence of the D816V KIT mutation and an elevated level of serum tryptase.

### Patients and data collection

For the mastocytosis patient cohort, all adult (≥18 years) patients suffering mastocytosis identified by AFIRMM between 1999 and 2004 were eligible. In September 2004, AFIRMM sent questionnaires to 703 of the identified adult patients. The patients were asked to respond to (i) a unidimensional questionnaire on their overall perception of disability (overall patient assessment [OPA] questionnaire) and (ii) a multidimensional questionnaire containing 38 items on the patient's perception of disability from individual symptoms (AFIRMM questionnaire). Of the 703 patients that were sent questionnaires, 363 provided responses. At the same time or at a later date, the patients were also administered questionnaires to assess seven measurable parameters of disability (see “Measurable parameters of disability” below). All questionnaires were returned to and processed at the Service d'Hématologie, Hôpital Necker (Paris, France). The case-control cohort included 90 members of the medical staff or their family members that were unaffected by and with no family member affected by mastocytosis. Data from questionnaires were collected according to French privacy laws, and all parts of the study were approved by the Ethics Committee of the Hôpital Necker, Paris, France.

Of the 363 patients that responded, 262 were part of an ongoing pathophysiological study started in November 2003 by AFIRMM. The patients selected and enrolled in the pathophysiological study included (i) patients suffering CM as documented by a skin biopsy and without mast cells in other tissues and (ii) patients suffering from SM as documented by mast cell infiltration in a bone marrow and/or another internal organ (i.e., liver or gastrointestinal tract) with or without skin involvement. In addition, all patients had to be affiliated with a social security regimen or covered by insurance. The patients in the pathophysiological study were recruited by investigators following a consultation where the investigator verbally informed the patients about the aims and conditions of the study. The patient received a patient information sheet, and written informed consent was obtained prior to the initiation of all study procedures. For these 262 patients, the following were collected or performed during a medical visit: demographics, information related to the diagnosis of mastocytosis, previous data on organ involvement and KIT characterization, WHO classification, physical examination (weight and vital signs), clinical examination (cutaneous and systemic symptoms), biological examination (hematology, biochemistry laboratory tests, and serum tryptase level), radiological examination, blood samples, skin biopsy, and bone marrow aspirate and/or biopsy. For these patients, a confirmation of diagnosis was given by a centralized review of all previous clinical and histopathological assessments. All procedures in the pathophysiological study were conducted according to guidelines for Good Clinical Practice [8].

### OPA Score

The OPA score was determined from a unidimensional questionnaire, where the patients were asked, “How do you assess your disability in general (pain, general health status, impact on your life)? Grade 0, no disability; grade 1, light disability; grade 2, moderate disability; grade 3, severe disability; grade 4, intolerable disability.”

### AFIRMM Score

A mutidimensional questionnaire (see Supporting Information) was designed by AFIRMM to collect information on patients' perception of the severity of their symptoms and their impact on the quality of life. The questionnaire was designed on the basis of patient interviews between 1999 and 2004 to include the most commonly reported symptoms. There were a total of 38 specific symptoms in 12 categories (skin, allergy/flush/shock, gastrointestinal tract, rheumatology, asthenia, neurology/psychiatry, respiratory, urology, infection, hemorrhoidal inflammation, libido, and sweat). Each disability was assigned a grade between 0 and 4 (0, none; 1, light; 2, moderate; 3, severe; 4, intolerable). In addition, each disability grade was assigned a weighting of 1 to 5 to reflect the impact of the symptom's severity on the quality of life. The AFIRMM score was then calculated as follows:
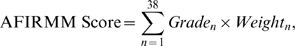
where n is the symptom number, *Grade* is the self-assessed severity of the symptom (0–4), and *Weight* = *Grade*+1. The resulting AFIRMM score can range from a minimum of 0 (no disability) to a maximum of 760 (most severe disability).

### Measurable parameters of disability

AFIRMM selected seven measurable parameters to confirm disability in mastocytosis. This included four quantifiable disabilities: existence of life-threatening anaphylactoid episodes, number of flushing episodes per week, number of stools per day, and number of micturitions per day. In addition, three scores used to measure disability in other pathologies were assessed: a pruritus score (see Supporting Information), the Hamilton score for depression [9], and the QLQ-C30 quality of life score [10]. For these measures, the following were considered as indicating a disability: the existence of recurrent life-threatening anaphylactoid episodes, ≥7 episodes of flushing per week, ≥4 stools per day (diarrhea), ≥8 micturitions per day (pollakiuria), pruritus score ≥6, Hamilton score ≥10 (depression), QLQ-C30 score ≥60.

### Detection of D816V KIT mutation

For patients with CM, biopsies were collected from skin lesions, and for patients with SM, bone marrow aspirates were collected from the sternum or iliac crest. Bone marrow samples (3 mL) were collected in an EDTA tube (Becton Dickinson), and skin samples consisted of two to three punch biopsies of 3 to 4 mm from a cutaneous lesion collected in 1 mL of RNAlater (Qiagen). All biopsy samples were sent to Fabienne Palmerini, Institut Paoli Calmettes (Marseille, France) at room temperature. Samples arrived within 36 to 60 h. Immediately after reception, skin samples were frozen at −80°C, and prior to RNA extraction, they were homogenized using a Polytron T25 Ultra-turrax (Fisher Bioblock Scientific) in RLT buffer at room temperature. Marrow samples were mixed with NH_4_Cl lysis solution (8.3 g/L NH_4_Cl, 0.81 g/L NaHCO_3_, and 0.37 g/L EDTA) and incubated for 10 min at 4°C to lyse red blood cells. The marrow cells were then sedimented by centrifugation at 180× g for 10 min, washed with phosphate-buffered saline, pH 7.4 (Gibco), resuspended in RLT buffer (Qiagen), and stored at −80°C for later RNA extraction.

Total RNA was extracted from thawed samples using an RNeasy Mini Kit (Qiagen). Complementary DNA (cDNA) was synthesized in a total volume of 50 µl containing 200 ng of total RNA, random hexamers, and oligo dT using the StrataScript first-strand synthesis system (Stratagene) according to the manufacturer's instructions. Next, 2.5 µl of cDNA was amplified by PCR using HotStartTaq™ DNA polymerase (Qiagen) and primers 5′-GGATGACGAGTTGGCCCTAGA-3′ and 5′-GTAGAAACTTAGATCGACCGGCA-3′. Amplification was carried out for 40 cycles at 94°C for 30 s, 57°C for 30 s, and 72°C for 45 s. PCR products were purified with the Geneclean III kit (Qbiogene) and directly sequenced using a BigDye terminator kit v1.1 (Applied Biosystems) with primers 5′-TACCAGGTGGCAAAGGGCATG-3′ and 5′-CGACCGGCATTCCAGGATAG-3′ on an ABI Prism 3130 sequencer (Applied Biosystems). The entire coding region for *KIT* was sequenced, and the obtained sequences were analyzed with Seqscape software (Applied Biosystems).

The presence of the D816V mutation was also confirmed by restriction digestion analysis using *Bsm*A1 and *Ple*1, which detect the wild-type and mutated form of *KIT*, respectively. The cDNA was amplified by PCR as described above but using fluorescently labeled primers. The size of restriction digest fragments (201 bp for the *Bsm*A1 fragment and 179 and 187 for the *Ple*1 fragment) were then directly determined on a 16-capillary sequencer (ABI Prism 3130) by comparison with Genescan rox 500 markers (Applied Biosystems) using GeneMapper software (Applied Biosystems).

### Measurement of serum tryptase

The level of total tryptase (α-protryptase+β-tryptase) in serum samples was determined using a fluorescence enzyme-linked immunoassay (Unicap; Pharmacia) [11]. The detection limit of this assay is 1 ng/mL, and in healthy controls, serum tryptase levels range between <1 and 15 ng/mL, with a median of ∼5 ng/mL [12].

### Statistical analyses

Statistical analyses were performed using SAS version 9.1 (SAS Institute) and Excel (Microsoft Corp.). Quantitative variables were summarized using the following descriptive statistics: number of observed and missing data, mean, standard deviation, median, minimum and maximum. Absolute and relative frequency distributions were provided for qualitative variables. Qualitative variables were compared using a Chi-square test or Fisher exact test if at least one of the expected counts was less than five. Normally distributed quantitative variables were compared using Student's *t*-test. Distributed quantitative variables that did not have a normal distribution were compared using Wilcoxon's rank sum test. *P*<0.05 was considered to indicate a statistically significant difference.

## Results

### Patient characteristics and data collected

**Figure 1:**
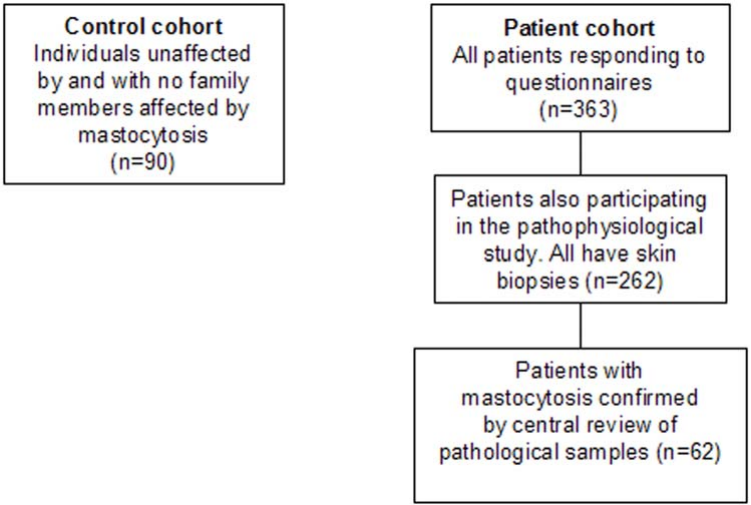
Control and patient groups in this study. A total of 363 patients and 90 controls answered AFIRMM and OPA questionnaires. Of the 363 patients, 262 were part of an ongoing pathophysiological study. Of these 262, 62 had their mastocytosis further validated by a careful centralized review of pathological samples and clinical data at Hôpital Necker.

During visits by mastocytosis patients between 1999 and 2004, we noticed that they tend to suffer from a very wide variety of common symptoms and that they often feel disabled by these symptoms. To gain a clearer picture of overall disability in mastocytosis and the type and severity of symptoms causing disability, we developed two questionnaires. The first was a unidimensional questionnaire asking the patients about their overall perception of disability (OPA questionnaire), and the second was a multidimensional questionnaire asking the patients about their perception of the severity of 38 symptoms in 12 categories (AFIRMM questionnaire; Table S1). In September 2004, we sent the questionnaires to 703 adult mastocytosis patients that had been identified by AFIRMM in France between 1999 and 2004. Responses to both questionnaires were obtained from 363 patients, 262 of whom were part of an ongoing pathophysiological study by AFIRMM. In addition, the questionnaires were administered to 90 control healthy subjects with no family members suffering from mastocytosis. The 363 patients included individuals with CM and both indolent and aggressive forms of SM, but none of the patients had mast cell leukemia (data not shown). Of the 262 patients in the pathophysiological study, 62 had their mastocytosis further validated by a central review of pathological samples and clinical data at Hôpital Necker (Figure 1).

### OPA Score

First, we compared the OPA scores between the patient and control cohorts (Table S2). The OPA score was based on patients' response to a single question: “How do you assess your disability in general (pain, overall health status, impact on your life)?” Of the 363 patients in the patient cohort, 254 (70%) declared that they suffered from a disability, including 62 (17%) who said it was severe or intolerable, whereas only 8 of 90 (9%) of the control patients said they had a disability (*P*<0.001 by Chi square test). Also, in contrast to the patients, none of the controls reported having a severe or intolerable disability.

We further examined the OPA score in the 262 patients in the pathophysiological study and the 62 patients whose mastocytosis was confirmed by a central review of pathological and clinical data at Hôpital Necker. Again, we found a significant difference in the fraction reporting a disability between the patients and controls. Thus, regardless of the level of validation, mastocytosis patients feel disabled by their disease.

We next examined whether the OPA score was different between patients with CM and SM (Table 1). Of the 262 patients for whom pathophysiological data was available, the WHO classification was known for 115. Of these, 33 (29%) were diagnosed with CM and the remaining 82 (71%) with SM. For patients diagnosed with CM, 64% (21/33) declared that they suffered from a disability, including 15% (5/33) who said that it was severe or intolerable, and of those with SM, 82% (67/82) reported having a disability and 28% (23/82) declared that it was severe or intolerable. The proportion declaring a disability was slightly different (*P* = 0.0386 by Chi square test) between patients with CM and SM; however, the difference was not significant when the analysis was limited to patients whose mastocytosis was further validated by a central review of all pathological and clinical data. These results indicate that CM and SM patients feel equally disabled by their disease.

**Table 1:** OPA scores.

*Variable*	Group	*n*	Perceived Handicap					
Population			None	Light	Moderate	Severe	Intolerable	*P*-value
*All cases*								
Controls		90	82 (91%)	5 (6%)	3 (3%)	0 (0%)	0 (0%)	
All patients		363	109 (30%)	107 (29%)	85 (23%)	59 (16%)	3 (1%)	*<0.0001* a
Patients in pathophysiological study		262	68 (26%)	75 (29%)	68 (26%)	49 (19%)	2 (1%)	*<0.0001* a
Patients confirmed by central review		62	8 (13%)	21 (34%)	16 (26%)	16 (26%)	1 (2%)	*<0.0001* a
*Clinical form*								
Patients in pathophysiological study	CM	33	12 (36%)	7 (21%)	9 (27%)	5 (15%)	0 (0%)	*0.0386* b
	SM	82	15 (18%)	28 (34%)	16 (20%)	22 (27%)	1 (1%)	
Patients confirmed by central review	CM	13	2 (15%)	3 (23%)	6 (46%)	2 (15%)	0 (0%)	0.61^c^
	SM	44	4 (9%)	15 (34%)	10 (23%)	14 (32%)	1 (2%)	
*KIT status*								
Patients in pathophysiological study	D816V	72	17 (24%)	19 (26%)	19 (26%)	16 (22%)	1 (1%)	0.64^d^
	No D816V	162	43 (27%)	52 (32%)	43 (27%)	23 (14%)	1 (1%)	
Patients confirmed by central review	D816V	16	2 (13%)	3 (19%)	6 (38%)	5 (31%)	0 (0%)	1.00^e^
	No D816V	40	6 (15%)	16 (40%)	10 (25%)	7 (18%)	1 (3%)	
*Serum tryptase*								
Patients in pathophysiological study	<20 ng/mL	53	12 (23%)	16 (30%)	13 (25%)	11 (21%)	1 (2%)	0.52^f^
	≥20 ng/mL	87	24 (28%)	24 (28%)	19 (22%)	20 (23%)	0 (0%)	
Patients confirmed by central review	<20 ng/mL	20	2 (10%)	7 (35%)	6 (30%)	4 (20%)	1 (5%)	0.70^g^
	≥20 ng/mL	34	6 (18%)	12 (35%)	7 (21%)	9 (26%)	0 (0%)	

a *P*-value calculated by Chi-square test for patients reporting a handicap vs. control.

b *P*-value calculated between CM and SM by Chi-square test.

c *P*-value calculated between CM and SM by Fisher's exact test.

d *P*-value calculated between presence and absence of D816V KIT mutation by Chi-square test.

e *P*-value calculated between presence and absence of D816V KIT mutation by Fisher's exact test.

f *P*-value calculated between serum tryptase ≤20 and >20 ng/mL by Chi-square test.

g *P*-value calculated between serum tryptase ≤20 and >20 ng/mL by Fisher exact test. Statistically significant differences (<0.05) are shown in italics

Because the KIT D816V mutation is suggested to correlate with the occurrence of SM in adult patients and to contribute to its progression [1], [13], we next examined whether it correlates with the OPA score (Table 1). The KIT status was known for 234 patients and the serum tryptase level for 140 of the patients. There was no difference in the OPA score according to the presence of the D816V KIT mutation for all patients whose classification was known or for patients whose mastocytosis was confirmed by a central review of pathological and clinical data. Collectively, these results indicate that patients' overall perception of disability is unrelated to the presence of the D816V mutation.

We also examined the correlation between the OPA score and the total serum tryptase level because a level ≥20 ng/mL is used as a criterion for a diagnosis SM [2], [14], [15]. In addition, recent studies show that the serum tryptase level positively correlates with the severity category of SM [16], [17]. As shown in Table 1, there was no significant difference between the OPA score according to the presence of an elevated (≥20 ng/mL) serum tryptase level. This was also found when the analysis was limited to patients whose mastocytosis was validated by a central review of pathological and clinical data. These findings indicate that the patients' overall perception of disability is unrelated to whether they have an elevated serum tryptase level.

### AFIRMM score

To allow analysis of the severity of the individual symptoms and their contribution to the patients' perception of disability in mastocytosis, we designed a questionnaire that assesses the severity or self-perceived severity of 38 distinct symptoms in 12 different categories. Each symptom was given a grade from 0 to 4, and each grade was assigned a weight from 1 to 5 to reflect the impact of each level of disability on the quality of life. This information was used to calculate a composite score (AFIRMM score) that ranged from 0 to 760 (from least to most severe).

As shown in Table 2, the mean AFIRMM score was significantly higher for the patient cohort than for the control cohort (mean±standard deviation, 117±78 [n = 363] vs. 29±27 [n = 90]; *P*<0.0001). This result was also found when the analysis was limited to the 262 patients who were part of the pathophysiological study and to the subsets of patients whose mastocytosis was validated by a central review of pathological and clinical data.

**Table 2:** AFIRMM scores.

*Variable*	Group	*n*	Mean±SD	Median	Min–Max	*P*-value
Population						
*All cases*						
Controls		90	29±27	21	0–150	–
All patients		363	117±84	104		*<0.0001* a
Patients in pathophysiological study		262	124.8±79.9	111	6.0–410.0	*<0.0001* a
Patients confirmed by central review		62	144.5±83.3	133	22.0–410.0	*<0.0001* a
*Clinical form*						
Patients in pathophysiological study	CM	33	103±75	84	20–408	*0.0225* b
	SM	82	135±82	124	6–410	
Patients confirmed by central review	CM	13	145.7±95.0	128	46.0–408.0	0.54^b^
	SM	44	152.9±79.0	144	50.0–410.0	
*KIT status*						
Patients in pathophysiological study	D816V	72	129±79	119	6–358	0.29^c^
	No D816V	162	119±79	104	6–410	
Patients confirmed by central review	D816V	16	136.0±65.2	130	28.0–240.0	0.96^c^
	No D816V	40	145.5±90.4	129	22.0–410.0	
*Serum tryptase*						
Patients in pathophysiological study	<20 ng/mL	53	117±75	116	26–410	0.86^d^
	≥20 ng/mL	87	121±82	102	6–408	
Patients confirmed by central review	<20 ng/mL	20	139.6±91.1	121	28.0–410.0	0.99^d^
	≥20 ng/mL	34	137.6±83.5	128	22.0–408.0	

a *P*-value for patient group vs. control cohort by Wilcoxson test.

b *P*-value for CM vs. SM by Wilcoxson test.

c *P*-values for D816V vs. no D816V by Wilcoxson test.

d *P*-value for serum tryptase ≤20 ng/mL vs. >20 ng/mL by Wilcoxson test. Statistically significant differences (<0.05) are shown in italics.

We found a slight difference in the AFIRMM score between patients with CM and SM (mean±standard deviation, 103±75 [n = 33] vs. 135±82 [n = 90]; *P* = 0.0225), but the difference was not significant when the analysis was limited to patients whose mastocytosis was validated by a central review of pathological and clinical data (Table 2). There was also not a significant difference between the AFIRMM score according to the presence or absence of the KIT D816V mutation or a serum tryptase level >20 ng/mL, regardless of the subgroup of patients analyzed. These results agree with the findings from the OPA score that mastocytosis patients feel disabled by their symptoms regardless of whether they have CM or SM, that the overall extent of disability does not differ significantly between these two main types of mastocytosis, and that overall disability is unrelated to the presence of the presence of the D816V mutation or an elevated serum tryptase level. Thus, the AFIRMM score appears to reflect the patients' overall perception of disability from mastocytosis.

We next examined the results on a symptom-by-symptom basis. We found that for the vast majority (32/38) of symptoms, there was a statistically significant difference in the proportion of patients and controls reporting some disability (grades 1–4) (Table 3). According to the score (*Grade*×*Weight*), the 10 symptoms most contributing to the patients' perception of disability were (in decreasing order) psychological impact of cutaneous problems, asthenia (fatigue), pruritus, food allergy/intolerance, erythemateous crisis, muscle and joint pain/cramps, pollakiuria, drug allergy, aerophagia/eruction, and dyspnea/bronchoreactivity. When the analysis was limited to patients whose mastocytosis was validated by a central review, there was some variability in whether certain symptoms (i.e., aerophagia/eruction, ocular discomfort, erectile function/ability to make love, and dysuria) were statistically significant or not (data not shown); however, except for dysuria, when only severe or intolerable disabilities (grades 3 or 4) were considered, all of these were found to be significantly more common in patients than in controls (Table 3), suggesting that they represent disabling symptoms of mastocytosis.

**Table 3:** Disability by symptom: patients vs. controls.

Symptom	Rank^a^	Controls			Patients			*P*-value^b^	
		*n*	Any disability^c^	Severe or intolerable disability^d^	*n*	Any disability^c^	Severe or intolerable disability^d^	Any disability^c^	Severe or intolerable disability^d^
Psychological impact	1	90	9 (10%)	1 (1%)	363	261 (72%)	120 (33%)	*<0.0001*	*<0.0001*
Asthenia	2	90	34 (38%)	3 (3%)	362	296 (82%)	102 (28%)	*<0.0001*	*<0.0001*
Pruritus	3	90	25 (28%)	3 (3%)	363	299 (82%)	82 (23%)	*<0.0001*	*<0.0001*
Food allergy/intolerance	4	90	9 (10%)	0 (0%)	363	222 (61%)	97 (27%)	*<0.0001*	*<0.0001*
Erythemateous crisis	5	90	17 (19%)	1 (1%)	363	293 (81%)	69 (19%)	*<0.0001*	*<0.0001*
Muscle and joint pain, cramps	6	90	36 (40%)	3 (3%)	363	276 (76%)	71 (20%)	*<0.0001*	*0.0002*
Pollakiuria	7	90	58 (64%)	6 (7%)	362	263 (73%)	64 (18%)	0.12	*0.0098*
Drug allergy	8	90	16 (18%)	0 (0%)	363	205 (56%)	70 (19%)	*<0.0001*	*<0.0001*
Aerophagia/eructation	9	90	43 (48%)	1 (1%)	363	229 (63%)	62 (17%)	*0.0080*	*<0.0001*
Dyspnea/bronchoreactivity	10	90	15 (17%)	3 (3%)	362	154 (43%)	94 (26%)	*<0.0001*	*<0.0001*
Headache	11	90	34 (38%)	4 (4%)	362	250 (69%)	48 (13%)	*<0.0001*	*0.0190*
Bone pain	12	90	16 (18%)	0 (0%)	363	196 (54%)	65 (18%)	*<0.0001*	*<0.0001*
Reduced sexual relations	13	90	11 (12%)	4 (4%)	362	132 (36%)	65 (18%)	*<0.0001*	*0.0014*
Epigastric pain	14	90	35 (39%)	2 (2%)	362	249 (69%)	40 (11%)	*<0.0001*	*0.0100*
Ocular discomfort	15	90	43 (48%)	1 (1%)	363	219 (60%)	55 (15%)	*0.0309*	*0.0003*
Memory loss	16	90	32 (36%)	0 (0%)	362	240 (66%)	34 (9%)	*<0.0001*	*0.0025*
Tinnitus	17	90	29 (32%)	1 (1%)	363	166 (46%)	47 (13%)	*0.0205*	*0.0011*
Pseudo-occlusive syndrome	18	90	20 (22%)	0 (0%)	363	199 (55%)	36 (10%)	*<0.0001*	*0.0018*
Infections (bronchitis, rhinitis, conjunctivitis)	19	90	25 (28%)	2 (2%)	363	182 (50%)	38 (10%)	*0.0001*	*0.0136*
Olfactive intolerance	20	90	33 (37%)	1 (1%)	363	188 (52%)	39 (11%)	*0.0102*	*0.0039*
Social interaction	21	90	9 (10%)	0 (0%)	362	200 (55%)	26 (7%)	*<0.0001*	*0.0088*
Depression	22	90	19 (21%)	0 (0%)	362	205 (57%)	22 (6%)	*<0.0001*	*0.0114* e
Mobility	23	90	6 (7%)	0 (0%)	363	153 (42%)	35 (10%)	*<0.0001*	*0.0022*
Anaphylactic shock	24	90	12 (13%)	0 (0%)	363	160 (44%)	30 (8%)	*<0.0001*	*0.0048*
Sweating	25	90	19 (21%)	2 (2%)	363	169 (47%)	30 (8%)	*<0.0001*	*0.0452*
Stomatitis	26	90	28 (31%)	1 (1%)	363	145 (40%)	34 (9%)	0.12	*0.0086*
Flush	27	90	9 (10%)	0 (0%)	363	190 (52%)	23 (6%)	*<0.0001*	*0.0123* e
Performance status	28	90	11 (12%)	1 (1%)	362	187 (52%)	25 (7%)	*<0.0001*	*0.0346*
Hemorrhoids	29	90	19 (21%)	1 (1%)	363	156 (43%)	23 (6%)	*0.0001*	0.06^e^
Cough	30	90	22 (24%)	0 (0%)	362	171 (47%)	9 (2%)	*<0.0001*	0.22^e^
Ear/nose/throat inflammation	31	90	13 (14%)	0 (0%)	362	120 (33%)	20 (6%)	*0.0005*	*0.0188* e
Erectile function/ability to make love	32	90	10 (11%)	2 (2%)	362	71 (20%)	34 (9%)	0.06	*0.0246*
Nausea, vomiting	33	90	20 (22%)	0 (0%)	363	179 (49%)	12 (3%)	*<0.0001*	0.14^e^
Diarrhea	34	90	6 (7%)	0 (0%)	363	127 (35%)	10 (3%)	*<0.0001*	0.22^e^
Warts	35	90	14 (16%)	1 (1%)	363	82 (23%)	10 (3%)	0.14	0.70^e^
Pain	36	90	4 (4%)	0 (0%)	362	71 (20%)	7 (2%)	*0.0005*	0.35^e^
Folliculitis	37	90	7 (8%)	1 (1%)	362	56 (15%)	6 (2%)	0.06	1.00^e^
Dysuria	38	90	6 (7%)	0 (0%)	362	51 (14%)	3 (1%)	0.06	1.00^e^

a Symptoms were ranked according to the average score (grade×weight).

b *P*-values were calculated between controls and patients by Chi square test except where noted.

c Grades 1–4.

d Grades 3 and 4 only.

e *P*-value calculated by Fisher's exact test. Statistically significant differences (<0.05) are shown in italics.

For the vast majority of individual symptoms, perceived disability did not differ between CM and SM patients (Table 4). Reduced erections or ability to make love was the only exception: a disability was reported by more SM than CM patients (31% vs. 3%; *P* = 0.0013), and a substantial portion (15%) of SM patients but none of the CM patients (*P* = 0.0176) reported this symptom as severe or intolerable. Also, slightly more SM patients reported disability from pseudo-occlusive syndrome (57% vs. 33%; *P* = 0.0200) and muscle/joint pain and cramps (88% vs. 70%; *P* = 0.0205), although the frequencies were not significantly different when only severe or intolerable disabilities (grades 3 or 4) were considered.

**Table 4:** Disability by symptom: comparison by classification (CM vs. SM).

Symptom	CM			SM			*P*-value^a^	
	*n*	Any disability^b^	Severe or intolerable disability^c^	*n*	Any disability^b^	Severe or intolerable disability^c^	Any disability^b^	Severe or intolerable disability^c^
Psychological impact	33	27 (82%)	12 (36%)	82	61 (74%)	33 (40%)	0.40	0.70
Asthenia	33	25 (76%)	9 (27%)	81	68 (84%)	29 (36%)	0.31	0.38
Pruritus	33	26 (79%)	6 (18%)	82	69 (84%)	23 (28%)	0.49	0.27
Food allergy/intolerance	33	19 (58%)	7 (21%)	82	52 (63%)	19 (23%)	0.56	0.82
Erythemateous crisis	33	26 (79%)	6 (18%)	82	70 (85%)	20 (24%)	0.39	0.47
Muscle and joint pain, cramps	33	23 (70%)	7 (21%)	82	72 (88%)	23 (28%)	*0.0205*	0.45
Pollakiuria	33	21 (64%)	5 (15%)	81	60 (74%)	20 (25%)	0.27	0.26
Drug allergy	33	19 (58%)	3 (9%)	82	48 (59%)	17 (21%)	0.92	0.14
Aerophagia/eructation	33	19 (58%)	6 (18%)	82	52 (63%)	13 (16%)	0.56	0.76
Dyspnea/bronchoreactivity	33	14 (42%)	8 (24%)	81	37 (46%)	24 (30%)	0.75	0.56
Headache	33	24 (73%)	5 (15%)	81	58 (72%)	9 (11%)	0.90	0.54^d^
Bone pain	33	18 (55%)	7 (21%)	82	58 (71%)	17 (21%)	0.10	0.95
Reduced sexual relations	33	7 (21%)	3 (9%)	82	41 (50%)	20 (24%)	*0.0046*	0.06
Epigastric pain	33	22 (67%)	2 (6%)	82	50 (61%)	8 (10%)	0.57	0.72^d^
Ocular discomfort	33	20 (61%)	5 (15%)	82	47 (57%)	10 (12%)	0.75	0.76^d^
Memory loss	33	21 (64%)	1 (3%)	81	58 (72%)	15 (19%)	0.40	*0.0366* d
Tinnitus	33	16 (48%)	5 (15%)	82	39 (48%)	15 (18%)	0.93	0.69
Pseudo-occlusive syndrome	33	11 (33%)	1 (3%)	82	47 (57%)	9 (11%)	*0.0200*	0.28^d^
Infections (bronchitis, rhinitis, conjunctivitis)	33	14 (42%)	2 (6%)	82	42 (51%)	10 (12%)	0.39	0.50^d^
Olfactive intolerance	33	17 (52%)	1 (3%)	82	48 (59%)	8 (10%)	0.49	0.44^d^
Social interaction	33	14 (42%)	1 (3%)	81	46 (57%)	7 (9%)	0.16	0.43^d^
Depression	33	15 (45%)	3 (9%)	81	51 (63%)	6 (7%)	0.09	0.72^d^
Mobility	33	14 (42%)	2 (6%)	82	47 (57%)	13 (16%)	0.15	0.23^d^
Anaphylactic shock	33	16 (48%)	3 (9%)	82	39 (48%)	8 (10%)	0.93	1.00^d^
Sweat	33	17 (52%)	3 (9%)	82	34 (41%)	8 (10%)	0.33	1.00^d^
Stomatitis	33	11 (33%)	3 (9%)	82	33 (40%)	10 (12%)	0.49	0.75^d^
Flush	33	23 (70%)	2 (6%)	82	47 (57%)	7 (9%)	0.22	1.00^d^
Performance status	33	16 (48%)	3 (9%)	81	53 (65%)	11 (14%)	0.09	0.75^d^
Hemorrhoids	33	10 (30%)	1 (3%)	82	40 (49%)	10 (12%)	0.07	0.17^d^
Cough	33	15 (45%)	0 (0%)	81	43 (53%)	2 (2%)	0.46	1.00^d^
Ear/nose/throat inflammation	33	11 (33%)	1 (3%)	81	24 (30%)	5 (6%)	0.70	0.67^d^
Erectile function/ability to make love	33	1 (3%)	0 (0%)	81	25 (31%)	12 (15%)	*0.0013*	*0.0176*
Nausea, vomiting	33	15 (45%)	2 (6%)	82	48 (59%)	3 (4%)	0.20	0.62^d^
Diarrhea	33	11 (33%)	0 (0%)	82	29 (35%)	6 (7%)	0.84	0.18^d^
Warts	33	6 (18%)	0 (0%)	82	16 (20%)	4 (5%)	0.87	0.32^d^
Pain	33	5 (15%)	0 (0%)	81	17 (21%)	3 (4%)	0.47	0.56^d^
Folliculitis	33	5 (15%)	1 (3%)	81	10 (12%)	1 (1%)	0.76	0.50^d^
Dysuria	33	4 (12%)	0 (0%)	81	15 (19%)	1 (1%)	0.41	1.00^d^

a *P*-values were calculated between CM and SM by Chi square test except where noted.

b Grades 1–4.

c Grades 3 and 4 only.

d *P*-value calculated by Fisher's exact test. Symptoms are listed in the same order as in Table 4. Statistically significant differences (<0.05) are shown in italics.

Comparison of the extent of disability from the individual symptoms according to the presence or absence of the D816V mutation also revealed few substantial differences (Table 5). Three of the 38 symptoms (erythemateous crisis, psychological impact, and pseudo-occlusive syndrome) were more common in those with the D816V mutation, and five symptoms (olfactive intolerance, gastric pain, mobility, ocular disorders, and stomatitis) were more common in those without the mutation. Of these, only the psychological impact of cutaneous problems was significantly different (more common in those with the D816V mutation) when only severe or intolerable disabilities were considered.

**Table 5:** Disability by symptom: comparison by the presence or absence of D816V KIT mutation.

Symptom	No D816V			D816V			*P*-value^a^	
	*n*	Any disability^b^	Severe or intolerable disability^c^	*n*	Any disability^b^	Severe or intolerable disability^c^	Any disability^b^	Severe or intolerable disability^c^
Psychological impact	72	41 (57%)	18 (25%)	162	133 (82%)	71 (44%)	*<0.0001*	*0.0062*
Asthenia	72	62 (86%)	26 (36%)	161	133 (83%)	45 (28%)	0.50	0.21
Pruritus	72	64 (89%)	18 (25%)	162	132 (81%)	42 (26%)	0.16	0.88
Food allergy/intolerance	72	46 (64%)	17 (24%)	162	95 (59%)	42 (26%)	0.45	0.71
Erythemateous crisis	72	52 (72%)	11 (15%)	162	140 (86%)	35 (22%)	*0.0090*	0.26
Muscle and joint pain, cramps	72	55 (76%)	21 (29%)	162	127 (78%)	28 (17%)	0.73	*0.0392*
Pollakiuria	72	47 (65%)	13 (18%)	161	120 (75%)	32 (20%)	0.15	0.75
Drug allergy	72	43 (60%)	15 (21%)	162	92 (57%)	31 (19%)	0.68	0.76
Aerophagia/eructation	72	50 (69%)	16 (22%)	162	94 (58%)	29 (18%)	0.10	0.44
Dyspnea/bronchoreactivity	72	34 (47%)	23 (32%)	161	68 (42%)	44 (27%)	0.48	0.47
Headache	72	53 (74%)	11 (15%)	161	108 (67%)	21 (13%)	0.32	0.65
Bone pain	72	41 (57%)	14 (19%)	162	90 (56%)	27 (17%)	0.84	0.61
Reduced sexual relations	72	29 (40%)	16 (22%)	161	62 (39%)	31 (19%)	0.80	0.60
Epigastric pain	72	53 (74%)	12 (17%)	162	97 (60%)	15 (9%)	*0.0432*	0.10
Ocular discomfort	72	52 (72%)	12 (17%)	162	89 (55%)	27 (17%)	*0.0126*	1.00
Memory loss	72	47 (65%)	6 (8%)	161	107 (66%)	19 (12%)	0.86	0.43
Tinnitus	72	33 (46%)	9 (13%)	162	80 (49%)	22 (14%)	0.62	0.82
Pseudo-occlusive syndrome	72	32 (44%)	7 (10%)	162	95 (59%)	16 (10%)	*0.0442*	0.97
Infections (bronchitis, rhinitis, conjunctivitis)	72	39 (54%)	8 (11%)	162	75 (46%)	18 (11%)	0.27	1.00
Olfactive intolerance	72	45 (63%)	10 (14%)	162	75 (46%)	12 (7%)	*0.0221*	0.12
Social interaction	72	41 (57%)	8 (11%)	161	87 (54%)	11 (7%)	0.68	0.27
Depression	72	41 (57%)	6 (8%)	161	99 (61%)	14 (9%)	0.51	0.93
Mobility	72	40 (56%)	9 (13%)	162	67 (41%)	15 (9%)	*0.0442*	0.45
Anaphylactic shock	72	40 (56%)	7 (10%)	162	72 (44%)	14 (9%)	0.12	0.79
Sweat	72	39 (54%)	10 (14%)	162	74 (46%)	13 (8%)	0.23	0.16
Stomatitis	72	36 (50%)	10 (14%)	162	58 (36%)	13 (8%)	*0.0409*	0.16
Flush	72	41 (57%)	4 (6%)	162	97 (60%)	11 (7%)	0.67	1.00^d^
Performance status	72	44 (61%)	6 (8%)	161	83 (52%)	13 (8%)	0.18	0.95
Hemorrhoids	72	31 (43%)	5 (7%)	162	76 (47%)	12 (7%)	0.58	0.90
Cough	72	40 (56%)	2 (3%)	161	68 (42%)	6 (4%)	0.06	1.00^d^
Ear/nose/throat inflammation	72	26 (36%)	5 (7%)	161	53 (33%)	6 (4%)	0.63	0.32^d^
Erectile function/ability to make love	72	16 (22%)	7 (10%)	161	34 (21%)	16 (10%)	0.85	0.96
Nausea, vomiting	72	43 (60%)	4 (6%)	162	79 (49%)	7 (4%)	0.12	0.74^d^
Diarrhea	72	33 (46%)	2 (3%)	162	53 (33%)	5 (3%)	0.05	1.00^d^
Warts	72	19 (26%)	2 (3%)	162	31 (19%)	5 (3%)	0.21	1.00^d^
Pain	72	17 (24%)	2 (3%)	161	28 (17%)	3 (2%)	0.27	0.65^d^
Folliculitis	72	12 (17%)	1 (1%)	161	22 (14%)	3 (2%)	0.55	1.00^d^
Dysuria	72	9 (13%)	1 (1%)	161	22 (14%)	0 (0%)	0.81	0.31^d^

a *P*-value calculated between CM and SM by Chi square test except where noted.

b Grades 1–4.

c Grades 3 and 4 only.

d *P*-value calculated by Fisher's exact test. Symptoms are listed in the same order as in Table 4. Statistically significant differences (<0.05) are shown in italics.

The extent of disability for the individual symptoms did not appear to differ according to the presence or absence of an elevated (>20 ng/mL) serum tryptase level (Table 6). Ocular discomfort was somewhat more common in patients with a serum tryptase level ≤20 ng/mL, but this difference was not observed when only severe or intolerable disabilities were considered.

**Table 6:** Disability by symptom: comparison by serum tryptase level.

Symptom	≤20 ng/ml			>20 ng/ml			*P*-value^a^	
	*n*	Any disability^b^	Severe or intolerable disability^c^	*n*	Any disability^b^	Severe or intolerable disability^c^	Any disability^b^	Severe or intolerable disability^c^
Psychological impact	53	34 (64%)	13 (25%)	87	67 (77%)	41 (47%)	0.10	*0.0077*
Asthenia	53	46 (87%)	20 (38%)	86	67 (78%)	27 (31%)	0.19	0.44
Pruritus	53	42 (79%)	13 (25%)	87	71 (82%)	19 (22%)	0.73	0.71
Food allergy/intolerance	53	32 (60%)	12 (23%)	87	52 (60%)	18 (21%)	0.94	0.78
Erythemateous crisis	53	40 (75%)	8 (15%)	87	71 (82%)	20 (23%)	0.38	0.26
Muscle and joint pain, cramps	53	41 (77%)	13 (25%)	87	68 (78%)	19 (22%)	0.91	0.71
Pollakiuria	53	35 (66%)	10 (19%)	86	60 (70%)	17 (20%)	0.65	0.90
Drug allergy	53	33 (62%)	9 (17%)	87	47 (54%)	19 (22%)	0.34	0.49
Aerophagia/eructation	53	30 (57%)	4 (8%)	87	51 (59%)	16 (18%)	0.81	0.08
Dyspnea/bronchoreactivity	53	23 (43%)	16 (30%)	86	38 (44%)	27 (31%)	0.93	0.88
Headache	53	35 (66%)	7 (13%)	86	58 (67%)	8 (9%)	0.86	0.47
Bone pain	53	31 (58%)	10 (19%)	87	56 (64%)	19 (22%)	0.49	0.67
Reduced sexual relations	53	20 (38%)	7 (13%)	87	35 (40%)	20 (23%)	0.77	0.15
Epigastric pain	53	31 (58%)	2 (4%)	87	54 (62%)	11 (13%)	0.67	0.13^d^
Ocular discomfort	53	37 (70%)	8 (15%)	87	42 (48%)	11 (13%)	*0.0127*	0.68
Memory loss	53	36 (68%)	6 (11%)	86	58 (67%)	7 (8%)	0.95	0.56^d^
Tinnitus	53	28 (53%)	9 (17%)	87	38 (44%)	12 (14%)	0.29	0.61
Pseudo-occlusive syndrome	53	23 (43%)	4 (8%)	87	48 (55%)	7 (8%)	0.18	1.00^d^
Infections (bronchitis, rhinitis, conjunctivitis)	53	26 (49%)	4 (8%)	87	38 (44%)	9 (10%)	0.54	0.77^d^
Olfactive intolerance	53	33 (62%)	10 (19%)	87	41 (47%)	3 (3%)	0.08	*0.0047* d
Social interaction	53	28 (53%)	3 (6%)	86	41 (48%)	6 (7%)	0.55	1.00^d^
Depression	53	33 (62%)	4 (8%)	86	48 (56%)	6 (7%)	0.45	1.00^d^
Mobility	53	25 (47%)	5 (9%)	87	45 (52%)	12 (14%)	0.60	0.44
Anaphylactic shock	53	26 (49%)	5 (9%)	87	41 (47%)	6 (7%)	0.82	0.75^d^
Sweat	53	28 (53%)	5 (9%)	87	38 (44%)	8 (9%)	0.29	1.00^d^
Stomatitis	53	23 (43%)	7 (13%)	87	28 (32%)	9 (10%)	0.18	0.61
Flush	53	31 (58%)	6 (11%)	87	45 (52%)	6 (7%)	0.44	0.37^d^
Performance status	53	33 (62%)	2 (4%)	86	49 (57%)	10 (12%)	0.54	0.13^d^
Hemorrhoids	53	19 (36%)	2 (4%)	87	43 (49%)	9 (10%)	0.12	0.21^d^
Cough	53	27 (51%)	1 (2%)	86	39 (45%)	1 (1%)	0.52	1.00^d^
Ear/nose/throat inflammation	53	14 (26%)	3 (6%)	86	25 (29%)	4 (5%)	0.74	1.00^d^
Erectile function/ability to make love	53	5 (9%)	1 (2%)	86	19 (22%)	10 (12%)	0.06	0.05^d^
Nausea, vomiting	53	33 (62%)	3 (6%)	87	40 (46%)	2 (2%)	0.06	0.37^d^
Diarrhea	53	18 (34%)	2 (4%)	87	29 (33%)	4 (5%)	0.94	1.00^d^
Warts	53	13 (25%)	1 (2%)	87	15 (17%)	3 (3%)	0.30	1.00^d^
Pain	53	11 (21%)	0 (0%)	86	17 (20%)	1 (1%)	0.89	1.00^d^
Folliculitis	53	5 (9%)	0 (0%)	86	15 (17%)	3 (3%)	0.19	0.29^d^
Dysuria	53	8 (15%)	0 (0%)	86	10 (12%)	1 (1%)	0.55	1.00^d^

a *P*-value calculated between CM and SM by Chi square test except where noted.

b Grades 1–4.

c Grades 3 and 4 only.

d *P*-value calculated by Fisher's exact test. Symptoms are listed in the same order as in Table 4. Statistically significant differences (<0.05) are shown in italics.

### Standard measures of disability

At the same time or subsequent to completing the OPA and AFIRMM questionnaires, the patients were asked to respond to questionnaires to assess standard measures of disability. This included four quantifiable measures of disability (existence of life-threatening anaphalactoid episodes, number of flushes per week, number of stools per day, and number of micturitions per day) and three scores that have been used to assess disability in other diseases, namely, pruritus score, Hamilton score for depression [9], and QLQ-C30 score, which was designed to measure the quality of life in oncology patients [10]. Patients were considered to have a disability when they had recurrent life-threatening anaphylactoid episodes (19%), ≥7 episodes of flushing per week (66%), ≥4 stools per day (12%), ≥8 micturitions per day (32%), a pruritus score ≥6 (77%), a Hamilton rating score ≥10 (75%), or a QLQ-C30 score ≥60 (32%) (Table 7). According to these scores, 61% of all responding mastocytosis patients presented at least one disability. Furthermore, there were no significant differences in these seven parameters of disability by clinical form (CM vs. SM; Table 8), the presence or absence of the KIT D818V mutation (Table 9), or the presence or absence of a serum tryptase level ≥20 ng/mL (Table 10).

**Table 7:** Standard measures of disability.

Parameter	*n*	No. with handicap
Existence of recurrent life-threatening anaphylactoid episodes	153	29 (19%)
≥7 flushes per week	92	61 (66%)
≥4 stools per day	90	11 (12%)
≥8 micturitions per day	92	29 (32%)
Pruritus score ≥6	90	69 (77%)
Hamilton scale ≥10	88	66 (75%)
QLQ-C30 ≥60	124	40 (32%)

Data was collected from the 262 patients that participated in the pathophysiological study.

**Table 8:** Standard measures of disability: comparison by classification.

Parameter	CM		SM		*P*-value^a^
	*n*	No. with handicap	*n*	No. with handicap	
Existence of recurrent life-threatening anaphylactoid episodes	21	3 (14%)	61	10 (16%)	1.00
≥7 flushes per week	8	6 (75%)	28	19 (68%)	1.00
≥4 stools per day	7	2 (29%)	28	4 (14%)	1.00
≥8 micturitions per day	8	1 (13%)	28	8 (29%)	1.00
Pruritus score ≥6	8	7 (88%)	27	19 (70%)	1.00
Hamilton scale ≥10	8	7 (88%)	26	22 (85%)	1.00
QLQ-C30 ≥60	10	4 (40%)	34	14 (41%)	1.00

a *P*-value calculated by Fisher's exact test. Data was collected from the 262 patients that participated in the pathophysiological study.

**Table 9:** Standard measures of disability: comparison by KIT mutation status.

Parameter	No D816V		D816V		*P*-value
	*n*	No. with handicap	*n*	No. with handicap	
Existence of recurrent life-threatening anaphylactoid episodes	37	8 (22%)	102	16 (16%)	0.41^a^
≥7 flushes per week	18	13 (72%)	67	45 (67%)	0.68^a^
≥4 stools per day	18	2 (11%)	65	8 (12%)	1.00^b^
≥8 micturitions per day	18	5 (28%)	67	21 (31%)	0.77^a^
Pruritus score ≥6	18	13 (72%)	66	51 (77%)	0.76^b^
Hamilton scale ≥10	18	14 (78%)	63	48 (76%)	1.00^b^
QLQ-C30 ≥60	28	9 (32%)	82	23 (28%)	0.68^a^

a *P*-value calculated by Chi-square test.

b *P*-value calculated by Fisher's exact test.

**Table 10:** Standard measures of disability: comparison by serum tryptase level.

Parameter	≤20 ng/ml		>20 ng/ml		*P*-value
	*n*	No. with handicap	*n*	No. with handicap	
Existence of recurrent life-threatening anaphylactoid episodes	28	4 (14%)	63	9 (14%)	1.00^b^
≥7 flushes per week	15	12 (80%)	29	17 (59%)	0.16^a^
≥4 stools per day	15	2 (13%)	28	4 (14%)	0.27^b^
≥8 micturitions per day	15	5 (33%)	29	5 (17%)	0.74^b^
Pruritus score ≥6	14	10 (71%)	28	18 (64%)	0.45^b^
Hamilton scale ≥10	14	12 (86%)	27	19 (70%)	0.38^a^
QLQ-C30 ≥60	24	10 (42%)	36	11 (31%)	0.82^a^

a *P*-value calculated by Chi-square test.

b *P*-value calculated by Fisher's exact test. Data was collected from the 262 patients that participated in the pathophysiological study.

## Discussion

This study was the first large-scale, comprehensive analysis of perceived disability in mastocytosis patients. The study included 363 mastocytosis patients in France identified by the AFIRMM network along with 90 control participants. The patient cohort included a mixture of patients with CM and indolent and aggressive forms of SM but no patients with mast cell leukemia.

According to the OPA score, which was a unidimensional self-assessment of disability, a majority of mastocytosis patients (70%) feel that they suffer from a disability, whereas only a minority of the controls (9%) indicated having a disability. We further examined patients' self-reported disability using the AFIRMM questionnaire and score, which were designed to provide a more comprehensive analysis of perceived disabilities from mastocytosis. The AFIRMM scores confirmed that most mastocytosis patients feel disabled by their disease symptoms. This perception of disability corresponded with the finding that a majority of mastocytosis patients had at least one of the seven measurable disabilities including life-threatening anaphylactoid episodes, 7 or more episodes of flushing per week, 4 or more stools per day (diarrhea), 8 or more micturitions per day (pollakiuria), a pruritus score of 6 or more, a Hamilton rating score of 10 or more (depression), or a QLQ-C30 score of 60 or more.

Analysis of the severity for the 38 individual symptoms on the AFIRMM questionnaire suggested that mastocytosis patients suffer from a wide variety of symptoms: for at least 32 of the 38 symptoms, there was a significant difference between the number of patients and controls reporting a disability. Analysis of the scores (*Grade*×*Weight*) for each symptom revealed that the 10 symptoms that most contributed to the perception of disability were (in decreasing order) psychological impact, asthenia, pruritus, food allergy/intolerance, erythemateous crisis, muscle and joint pain/cramps, pollakiuria, drug allergy, aerophagia/eruction, and dyspnea/bronchoreactivity. Our results also confirmed the existence of number of previously described symptoms associated with mastocytosis, including fatigue (asthenia), anaphylaxis, sweating, flushing, pruritus, erythemateous crises, epigastric pain, diarrhea, nausea/vomiting, bone pain, headache, memory loss, difficulty with social interactions, reduced performance status, and depression [3], [17]. Importantly, our results reveal that the number of symptoms significantly associated with mastocytosis appears to be even larger than previously considered; for the first time, we provide strong evidence that mastocytosis patients also feel disabled by food and drug allergy/intolerance, muscle/joint pain and cramps, aerophagia/eruction, reduced sexual relations, ocular discomfort, tinnitus, pseudo-occlusive syndrome, infections (bronchitis, rhinitis, and conjunctivitis), olfactive intolerance, reduced mobility, hemorrhoidal inflammation, cough, ear/nose/throat inflammation, and general pain (Table 11).

**Table 11:** Symptoms significantly associated with mastocytosis according to AFIRMM score.

Previously identified symptoms^a^	Symptoms not previously reported
Fatigue (asthenia)	Food and drug allergy/intolerance
Anaphylaxis	Muscle/joint pain and cramps
Sweating	Aerophagia/eruction
Flushing	Reduced sexual relations
Pruritus	Ocular discomfort
Erythemateous crises	Tinnitus
Epigastric pain	Pseudo-occlusive syndrome
Diarrhea	Infections (bronchitis, rhinitis, and conjunctivitis)
Dyspnea/bronchoreactivity	Olfactive intolerance
Nausea/vomiting	Reduced mobility
Bone pain	Hemorrhoidal inflammation
Headache	Cough
Memory loss	Ear/nose/throat inflammation
Difficulty with social interactions	General pain
Reduced performance status	
Depression	

a Symptoms previously identified as described in references 3, 17, 20.

An important finding from this analysis was that psychological and neurological symptoms appear to be key contributors to disability in mastocytosis. We found that the psychological impact of the skin appearance was the symptom that most contributed to perception of disability in all mastocytosis patients: 72% of patients reported some disability due to this symptom, and 23% considered it severe or intolerable. Asthenia was another very common complaint, with 82% of mastocytosis patients reporting a disability, and a high proportion (28%) describing it as severe or intolerable. Also, patients reported suffering from reduced performance status (52%), difficulty with social interactions (55%), depression (57%), memory loss (66%), headache (69%), and pain (20%). That depression is a common symptom of mastocytosis was confirmed by the finding that more than 70% of all mastocytosis patients had a Hamilton score >10. Our observation that psychological and neurological effects are common in mastocytosis extends earlier findings by Rogers et al. [17], who found that SM is associated with diminished attention and memory, anger, irritability, and depression, a combination of symptoms that they referred to as an “atypical or mixed organic brain syndrome”. Also, a study in the late 1970s by Soter et al. [18] reported neuropsychiatric symptoms in five of eight mastocytosis patients, including poor attention span, irritability, fatigue, difficulty in concentrating, headache, inability to work effectively, problems in dealing with other people, and poor motivation. Overall, our results support the notion that the neurologic manifestations of SM are more common than previously thought [19], and they support the suggestion that mastocytosis patients may often be misdiagnosed due the nonspecificity of their neurologic symptoms [3].

In addition, our findings confirm that cutaneous, gastrointestinal, and skeletal symptoms are common to SM [3], [20]. We also found that pulmonary symptoms (i.e., cough and dyspnea/bronchoreactivity) are significantly associated with mastocytosis. In fact, 26% of all mastocytosis patients report suffering from severe or intolerable dyspnea or bronchoreactivity, and this symptom was the tenth most important contributor to overall disability according to the AFIRMM score. Another unexpected finding was that reduced sexual relations was one of the more important symptoms, with as many as 18% of patients describing it as severe or intolerable.

The key finding of this study was that the overall extent of disability and the type and severity symptoms were essentially the same between CM and SM patients. Initially, we found that patients' overall perception of disability as determined by both the OPA and AFIRMM scores does not depend on the clinical form of the disease. Importantly, we also found that there was no statistical difference between CM and SM in the seven standard measures of disability. These unexpected findings were confirmed when the analyses were limited to patients whose diagnosis of mastocytosis was validated by a careful centralized review of pathological and clinical data. On the basis of these results, we propose that CM and SM and their different subtypes are not distinct diseases but are part of a continuous spectrum of mast cell-related dysfunctions. Furthermore, this implies that the presence of excessive mast cells in extracutaneous tissues such as bone marrow is not helpful for understanding the disability and symptoms–real and/or perceived–of mastocytosis patients.

The KIT D816V mutation has been suggested to correlate with the occurrence of SM in adult patients and to contribute to its progression [1], [13]. However, there was no difference in disability as measured by OPA score, AFIRMM score, or measurable parameters of disability between patients with and without this mutation. Likewise, for 37 of the 38 symptoms, there was little or no difference in the fraction of patients reporting a disability according to the presence of the D816V mutation. Thus, the type and severity of mastocytosis symptoms appears to be unrelated to the presence of the D816V KIT mutation.

We also did not find a significant difference in the overall perception of disability (OPA and AFIRMM scores) or standard measures of disability according to the serum tryptase level (≤20 vs. >20 ng/mL). Likewise, the serum tryptase level appeared to have no bearing on the severity of the 38 individual symptoms as measured by the AFIRMM score or the standard measures. Because our assay measured total tryptase levels, which is an indicator of total mast cell burden [14], it appears that the type and severity of most symptoms are unrelated to elevated mast cell numbers. Accordingly, we expect that the level of activated, mature tryptase, which is a measure of mast cell activation [14], will be more useful than total serum tryptase for understanding the pathogenesis of mastocytosis.

### Conclusions

In summary, these results suggest that the existing classification system for mastocytosis does not help understand patients' overall perception of disability, their individual symptoms, or their impact on the quality of life. It appears that the presence of elevated mast cell numbers in bone marrow or extracutaneous tissues, an elevated serum tryptase level, and the D816V KIT mutation has little or no bearing on the type or severity symptoms. Given these results, we suspect that the symptoms of indolent mastocytosis are mostly unrelated to mast cell proliferation or infiltration; rather, it appears that the symptoms are the result of mast cell activation and the systemic release of mediators. On this basis, we propose an alternative classification system to help in the treatment of mastocytosis patients, namely, that (i) mast cell leukemia and aggressive mastocytosis be considered and treated separately from indolent forms and (ii) that indolent mastocytosis be classified and treated simply according to the patient's perception of disability due to symptoms of the disease.

Another important finding was that, although none of the symptoms are specific to the disease, the number associated with mastocytosis is even higher than previously realized and includes some symptoms that have been largely overlooked, especially neuropsychiatric symptoms. Given the wide variety and nonspecificity of these symptoms, we suspect that many mastocytosis patients are misdiagnosed and that the prevalence of mastocytosis is therefore higher than previously considered.

Finally, as part of the current study, we developed and validated a questionnaire and composite score for assessing patients' perception of disability in mastocytosis (AFIRMM score). This scoring system should be useful in evaluating the efficacy of mastocytosis treatments in future clinical trials and for further understanding the nature of symptoms and disability from mastocytosis.

## Supporting Information

Table S1 AFIRMM questionnaire
(0.14 MB DOC)

Table S2 Pruritus questionnaire and score
(0.04 MB DOC)
