# Supplementary material for: Case-Control Cohort Study of Patients' Perceptions of Disability in Mastocytosis
Source: PLoS One. 2008 May 28;3(5):e2266. doi: 10.1371/journal.pone.0002266 (PMC2386235; doi:10.1371/journal.pone.0002266)
Supplement: Table S1 — AFIRMM questionnaire (0.14 MB DOC) [file pone.0002266.s001.doc]

**Table S1.** AFIRMM questionnaire

| Category | No. | Handicap | Grade description | Grade | Weight | Points |
| --- | --- | --- | --- | --- | --- | --- |
| Skin | 1 | Pruritus (itching) | Not affected  Occasionally, sensitivity to 1 or 2 stimuli  Occasionally, sensitivity to more than 2 stimuli  Nearly all the time, but not during sleep  All the time, including during sleep | 0  1  2  3  4 | 1  2  3  4  5 | 0  2  6  12  20 |
|  | 2 | Erythemateous crisis (inflammation; getting red) | Not affected  Occasionally, sensitivity to 1 or 2 stimuli  Occasionally, sensitivity to 3 to 5 stimuli  Occasionally, sensitivity to 6 or more stimuli  All the time | 0  1  2  3  4 | 1  2  3  4  5 | 0  2  6  12  20 |
|  | 3 | Psychological impact | Not affected  Mild embarrassment to show one's body  Moderate embarrassment to show one's body  Severe embarrassment to show one's body  Body not shown any more | 0  1  2  3  4 | 1  2  3  4  5 | 0  2  6  12  20 |
| Allergy/ Flush/ Shock | 4 | Food allergy | Not affected  Less than 2 types of food withdrawn  2 to 4 types of food withdrawn  5 to 8 types of food withdrawn  9 or more types of food withdrawn, and shock if taken | 0  1  2  3  4 | 1  2  3  4  5 | 0  2  6  12  20 |
|  | 5 | Drug allergy | Not affected  Less than 2 types of medications withdrawn  2 to 4 types of medications withdrawn  5 to 8 types of medications withdrawn  9 or more types of medications withdrawn, and shock if taken | 0  1  2  3  4 | 1  2  3  4  5 | 0  2  6  12  20 |
|  | 6 | Olfactive intolerance (tobacco, perfume, etc.) | Not affected  Difficulty to stay in a room where there is intolerable smell  Must go out of a room where there is intolerable smell  Allergic reaction to an intolerable smell  Shock to an intolerable smell | 0  1  2  3  4 | 1  2  3  4  5 | 0  2  6  12  20 |
|  | 7 | Anaphylactic shock, syncope, dizziness | Not affected  1 – 2 in a year  3 – 6 in a year  7 – 12 in a year  13 or more in a year | 0  1  2  3  4 | 1  2  3  4  5 | 0  2  6  12  20 |
|  | 8 | Flush (redness, feeling of heat on the face) | Not affected  1 – 2 in a day  3 – 4 in a day  5 – 8 in a day  9 or more in a day | 0  1  2  3  4 | 1  2  3  4  5 | 0  2  6  12  20 |
| Gastro-intestinal track | 9 | Aerophagia, eructation | Not affected  Occasionally  Frequently  Nearly all the time  All the time | 0  1  2  3  4 | 1  2  3  4  5 | 0  2  6  12  20 |
|  | 10 | Nausea, vomiting | Not affected  Occasionally  Frequently  Nearly all the time  All the time | 0  1  2  3  4 | 1  2  3  4  5 | 0  2  6  12  20 |
|  | 11 | Epigastric pain | Not affected  Occasionally  Frequently  Nearly all the time  All the time | 0  1  2  3  4 | 1  2  3  4  5 | 0  2  6  12  20 |
|  | 12 | Diarrhea | Not affected  1 – 2 in a day  3 – 4 in a day  5 – 8 in a day  9 or more in a day | 0  1  2  3  4 | 1  2  3  4  5 | 0  2  6  12  20 |
|  | 13 | Pseudo-occlusive syndrome (distended abdomen, sometimes painful, with need for flatulence without success) | Not affected  Occasionally  Frequently  Nearly all the time  All the time | 0  1  2  3  4 | 1  2  3  4  5 | 0  2  6  12  20 |
| Rheumatol-ogy | 14 | Bone pain | Not affected  Mild pain  Moderate pain  Severe pain  Intolerable pain | 0  1  2  3  4 | 1  2  3  4  5 | 0  2  6  12  20 |
|  | 15 | Muscle and joint pain, cramps | Not affected  Mild pain  Moderate pain  Severe pain  Intolerable pain | 0  1  2  3  4 | 1  2  3  4  5 | 0  2  6  12  20 |
|  | 16 | Mobility | Not affected  Mildly affected for running  Cannot run but walks normally  Cannot run, affected for walking  Cannot walk, no mobility | 0  1  2  3  4 | 1  2  3  4  5 | 0  2  6  12  20 |
| Asthenia | 17 | Asthenia (fatigue) | Not affected  Mild  Moderate  Severe  Intolerable | 0  1  2  3  4 | 1  2  3  4  5 | 0  2  6  12  20 |
| Neurology/ Psychiatry | 18 | Performance status | Works normally  Works but needs rest  Doest not work anymore, must rest less than 50% of the day  Doest not work anymore, must rest more than 50% of the day  Needs permanent rest (bedridden) | 0  1  2  3  4 | 1  2  3  4  5 | 0  2  6  12  20 |
|  | 19 | Social interaction (irritability, resistance to stress) | Not affected  Mild difficulties to interact with others  Moderate difficulties to interact with others  Severe difficulties to interact with others  Cannot interact with the others | 0  1  2  3  4 | 1  2  3  4  5 | 0  2  6  12  20 |
|  | 20 | Depression | Not affected  Mild depression  Moderate depression  Severe depression  Severe depression with suicide attempt | 0  1  2  3  4 | 1  2  3  4  5 | 0  2  6  12  20 |
|  | 21 | Memory loss (ability to remember names or words) | Not affected  Mild  Moderate  Severe  Intolerable | 0  1  2  3  4 | 1  2  3  4  5 | 0  2  6  12  20 |
|  | 22 | Headache | Not affected  Mild  Moderate  Severe  Intolerable | 0  1  2  3  4 | 1  2  3  4  5 | 0  2  6  12  20 |
| Respiratory | 23 | Cough | Not affected  Intermittent  Frequent  Permanent but not during sleep  Permanent even during sleep | 0  1  2  3  4 | 1  2  3  4  5 | 0  2  6  12  20 |
|  | 24 | Dyspnea, asthma (breathing difficulties, shortness of breath) | Not affected  Intermittent asthma  Frequent asthma  Exercise dyspnea  Permanent dyspnea | 0  1  2  3  4 | 1  2  3  4  5 | 0  2  6  12  20 |
| Urology | 25 | Pollakiuria | Less than 5 micturations per day  5 – 6 micturations per day  7 – 8 micturations per day  9 – 10 micturations per day  More than 10 micturations per day | 0  1  2  3  4 | 1  2  3  4  5 | 0  2  6  12  20 |
|  | 26 | Pain | Not affected  Mild  Moderate  Severe  Intolerable | 0  1  2  3  4 | 1  2  3  4  5 | 0  2  6  12  20 |
|  | 27 | Dysuria (difficulties to urinate) | Not affected  Mild  Moderate  Severe  Impossible without tube | 0  1  2  3  4 | 1  2  3  4  5 | 0  2  6  12  20 |
| Infection | 28 | Folliculitis | Not affected  Mild  Moderate  Severe but enhanced with antibiotics  Severe and resistant to antibiotics | 0  1  2  3  4 | 1  2  3  4  5 | 0  2  6  12  20 |
|  | 29 | Ear/nose/throat inflammation | Not affected  Mild  Moderate  Severe  Intolerable | 0  1  2  3  4 | 1  2  3  4  5 | 0  2  6  12  20 |
|  | 30 | Tinnitus, hum | Never  With a triggering factor  Intermittent (less than one week)  More than once a day  At night, preventing sleep | 0  1  2  3  4 | 1  2  3  4  5 | 0  2  6  12  20 |
|  | 31 | Ocular discomfort (dry, red, or stinging eyes) | Never  Exceptionally with triggering factors  Often with triggering factors  Often, without triggering factor  Often, resistant to treatment | 0  1  2  3  4 | 1  2  3  4  5 | 0  2  6  12  20 |
|  | 32 | Stomatitis (gum inflammation or hemorrhage, tooth loss) | Never  Exceptionally with triggering factors  Often, with triggering factors  Often, without triggering factor  Resistant to treatment | 0  1  2  3  4 | 1  2  3  4  5 | 0  2  6  12  20 |
|  | 33 | Resistant warts, herpes | Not affected  Mild  Moderate  Severe  Intolerable | 0  1  2  3  4 | 1  2  3  4  5 | 0  2  6  12  20 |
|  | 34 | Infections (bronchitis, rhinitis, conjunctivitis) | Not affected less than once a year)  1 – 2 times a year  3 – 4 times a year  5 – 6 times a year  ≥ 7 times a year | 0  1  2  3  4 | 1  2  3  4  5 | 0  2  6  12  20 |
| Hemorrhoids | 35 | Hemorrhoids | Not affected  Mild  Moderate  Severe  Intolerable | 0  1  2  3  4 | 1  2  3  4  5 | 0  2  6  12  20 |
| Libido | 36 | Sexual relations (frequency of intercourse) | Not affected or not applicable  25% decrease  50% decrease  75% decrease  No sexual relations any more | 0  1  2  3  4 | 1  2  3  4  5 | 0  2  6  12  20 |
|  | 37 | Erectile function, possibility to have sexual relationship | Not affected or not applicable  Mild dysfunction  Moderate dysfunction  Severe dysfunction  Impossible | 0  1  2  3  4 | 1  2  3  4  5 | 0  2  6  12  20 |
| Sweat | 38 | Sweat | Not affected  Mild increase, controlled with cosmetics  Sharp increase, controlled with cosmetics  Sharp increase, uncontrolled with cosmetics  Intolerable for the others | 0  1  2  3  4 | 1  2  3  4  5 | 0  2  6  12  20 |
| Other symptoms (please describe) | 39 |  | No handicap  Mild handicap  Moderate handicap  Severe handicap  Intolerable handicap | 0  1  2  3  4 | 1  2  3  4  5 | 0  2  6  12  20 |
