# Supplementary material for: Case-Control Cohort Study of Patients' Perceptions of Disability in Mastocytosis
Source: PLoS One. 2008 May 28;3(5):e2266. doi: 10.1371/journal.pone.0002266 (PMC2386235; doi:10.1371/journal.pone.0002266)
Supplement: Table S2 — Pruritus questionnaire and score (0.04 MB DOC) [file pone.0002266.s002.doc]

Table S2. Pruritus questionnaire and score

| **ITEM** | **DEFINITION** | **GRADE** |
| --- | --- | --- |
| **Frequency of pruritus:** Pruritus is present | Every day | 1 |
|  | Every second day | 2 |
|  | Sporadically | 3 |
| **Intensity of pruritus** | Disabling | 1 |
|  | Significant | 2 |
|  | Moderate | 3 |
|  | Mild | 4 |
| **Localization** | Head | 1 |
|  | Back | 2 |
|  | Anterior surface of the trunk | 3 |
|  | One hand | 4 |
|  | Both hands | 5 |
|  | One leg | 6 |
|  | Both legs | 7 |
| **Influence on well-being** | Enormous | 1 |
|  | Moderate | 2 |
|  | Little | 3 |

The pruritus score was calculated as the total of the grades.
